# Impact of social media on self-esteem and body image among young adults

**DOI:** 10.1192/j.eurpsy.2022.1499

**Published:** 2022-09-01

**Authors:** R. Molina Ruiz, I. Alfonso-Fuertes, S. González Vives

**Affiliations:** 1 Hospital clínico san carlos, Psychiatry, Madrid, Spain; 2 Comillas University, Psychology, Madrid, Spain

**Keywords:** self esteem, social media, body image, Instagram

## Abstract

**Introduction:**

The extent to which social media contributes to body image dissatisfaction and lower self-esteem is currently under debate

**Objectives:**

This research seeks to study the relationship between the use of Instagram (one of the main platforms used by young people nowadays) and the degree of dissatisfaction with body image and the level of self-esteem among their younger users

**Methods:**

A sample of 585 Spanish adults between 18 and 40 years old was used, to whom the Body Shape Questionnaire (BSQ), the Rosenberg Self-esteem Scale and the Social Comparison of Appearance Scale (PACS) were applied.

**Results:**

A positive correlation was observed between the frequency of use of the social network and dissatisfaction with body image and low self-esteem. In addition, it was found that content observation time significantly predicts body dissatisfaction and low self-esteem. On the other hand, the type of content both published and observed, showed no effect on any of these variables, although it has been found that the relationship between the use of the platform and the study variables seems to be mediated by the tendency of their users to compare their appearance with others.

**Conclusions:**

These results invite us to reflect on the use of social networks and their impact on constructs as relevant to the person as self-esteem and body image and on how different policies should be taken into account to prevent a negative impact on the mental health of their users

**Disclosure:**

No significant relationships.

